# Supplementary figures and images for: Phylogenetic structure of moth communities (Geometridae, Lepidoptera) along a complete rainforest elevational gradient in Papua New Guinea
Source: PLoS One. 2024 Aug 12;19(8):e0308698. doi: 10.1371/journal.pone.0308698 (PMC11318904; doi:10.1371/journal.pone.0308698)

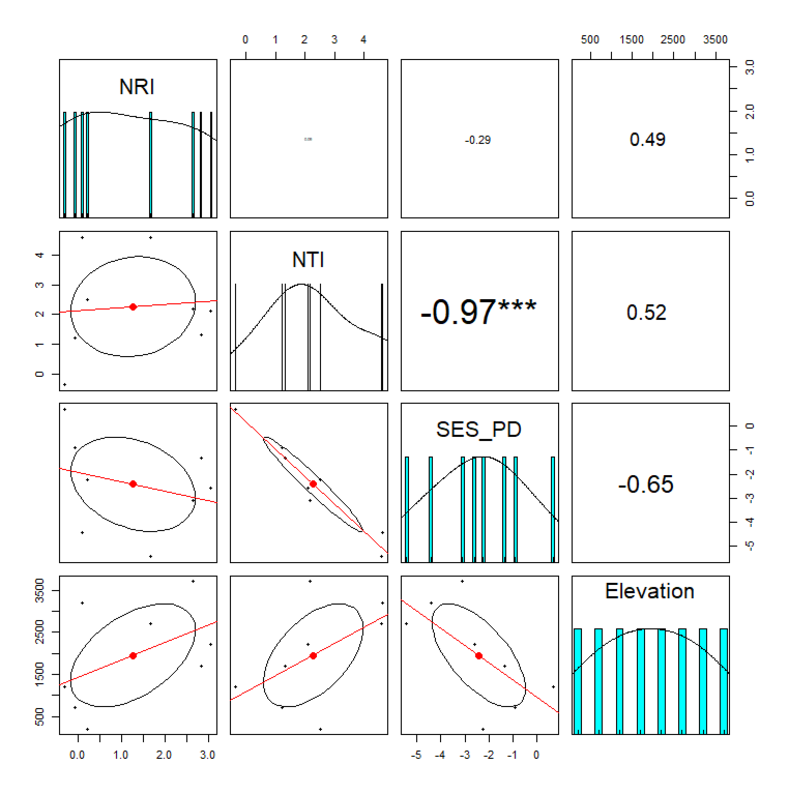

Supplement: S1 Fig — The x and y axis for each parameter is given according to the scale of measurements. Red lines are linear fit corresponding with the magnitude of correlations as shown by the correlation factors. (TIF) [file pone.0308698.s001.tif]

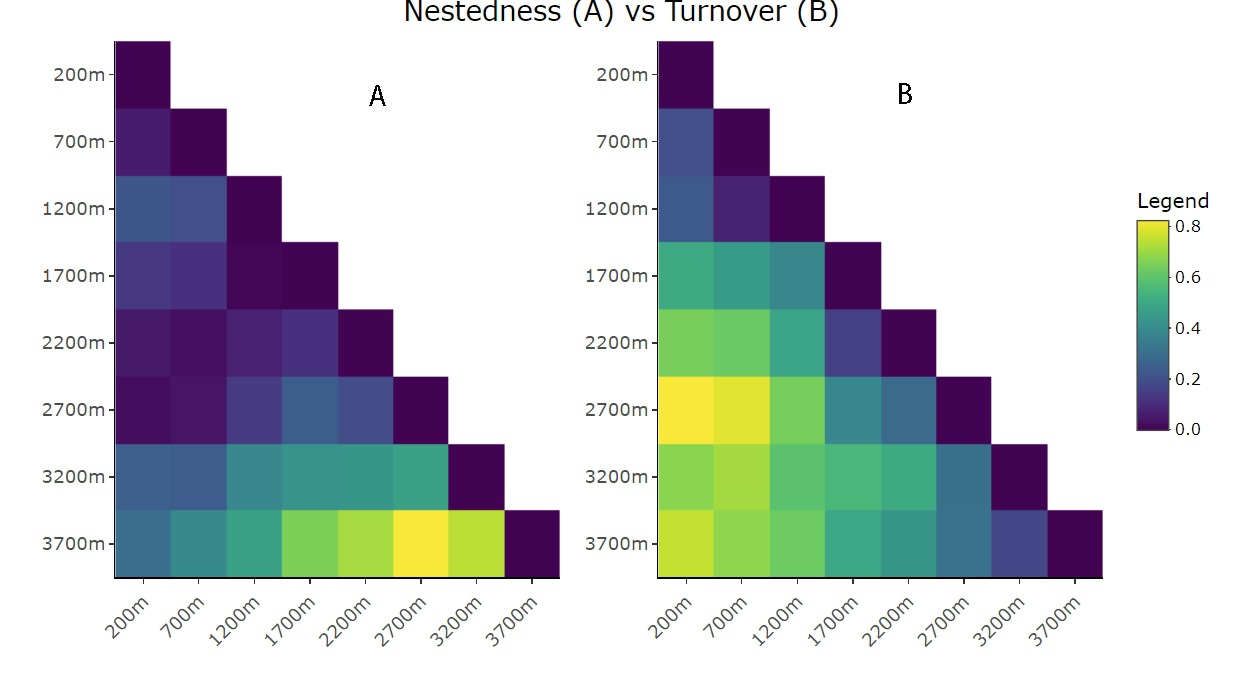

Supplement: S2 Fig — Phylogenetic beta-diversity of Geometridae moth communities along Mt. Wilhelm gradient showing nestedness (A) and turnover (B). Both indices range from 0 to 1: 0 depicts species community having no nestedness (A) or unique communities (B) and the value of 1 depicts communities with high nestedness (A) and high shared species in turnover (B). (TIF) [file pone.0308698.s002.tif]

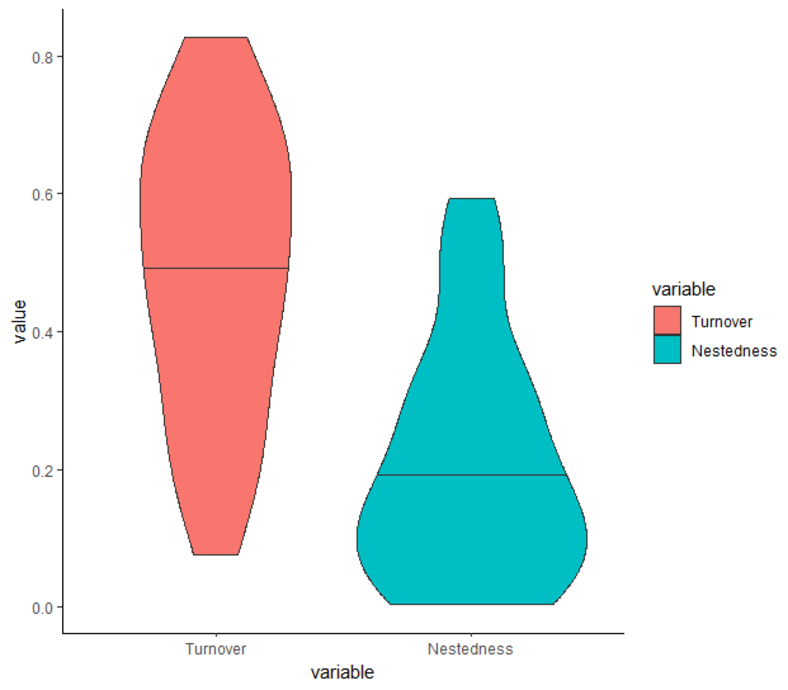

Supplement: S3 Fig — Turnover places more influence on the overall phylogenetic beta diversity. (TIF) [file pone.0308698.s003.tif]

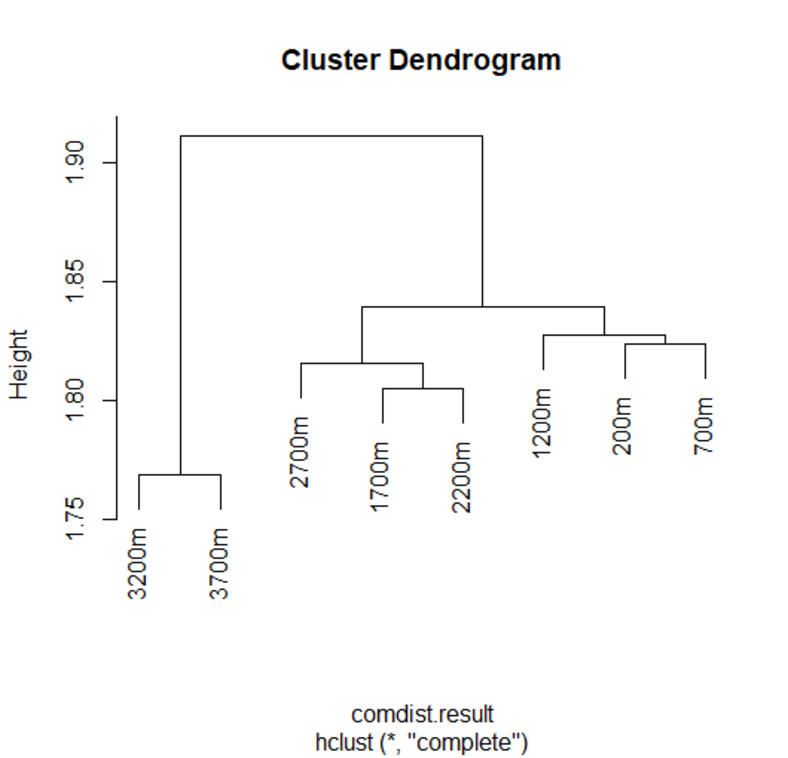

Supplement: S4 Fig — There are two principal clusters: one with highland communities formed by top two elevation sites (3200 and 3700 m a.s.l) and rest of the sites as lowland cluster further clustering to mid (2700, 2200 and 1700 m a.s.l) and lowland (1200, 700 and 200 m a.s.l) clusters relative to moth species genetic differences. (TIF) [file pone.0308698.s004.tif]
