# Supplementary material for: Phylogenetic structure of moth communities (Geometridae, Lepidoptera) along a complete rainforest elevational gradient in Papua New Guinea
Source: PLoS One. 2024 Aug 12;19(8):e0308698. doi: 10.1371/journal.pone.0308698 (PMC11318904; doi:10.1371/journal.pone.0308698)
Supplement: S1 Table — The observed values of the predictors are given here, and their standardized z-score (SES) values are given in Table 2 in the main text. * Indicates the data used for this study. (DOCX) [file pone.0308698.s005.docx]

**S1 Table:** Geometridae moth abundance, the number of morpho-species and the sequenced species occurrences per elevation. The observed values of the predictors are given here, and their standardized z-score (SES) values are given in Table 2 in the main text. * Indicates the data used for study.

|  | Moth data | | |  | Predictors | | |
| --- | --- | --- | --- | --- | --- | --- | --- |
| Elevation  (m a.s.l) | Abundance | Morpho spp. | Sequenced species occurrence* |  | plant.spp* | Pred.abun* | mean.temp* |
| 200 | 2,311 | 201 | 179 |  | 53 | 498 | 24.512 |
| 700 | 1,805 | 239 | 198 |  | 79 | 461 | 22.3 |
| 1200 | 2,194 | 391 | 283 |  | 82 | 386 | 19.477 |
| 1700 | 3,903 | 403 | 289 |  | 68 | 313 | 18.384 |
| 2200 | 2,631 | 305 | 232 |  | 41 | 277 | 15.655 |
| 2700 | 1,134 | 197 | 156 |  | 63 | 320 | 13.148 |
| 3200 | 2,091 | 182 | 45 |  | 55 | 227 | 9.878 |
| 3700 | 355 | 37 | 8 |  | 18 | 126 | 7.896 |
| Total | **16,424** | **1,955** | **1,390** |  | **459** | **2,608** | **16.406** |
