# Supplementary material for: Phylogenetic structure of moth communities (Geometridae, Lepidoptera) along a complete rainforest elevational gradient in Papua New Guinea
Source: PLoS One. 2024 Aug 12;19(8):e0308698. doi: 10.1371/journal.pone.0308698 (PMC11318904; doi:10.1371/journal.pone.0308698)
Supplement: S2 Table — The best model is the one with lowest AIC score at the top as generated from IQtree model selection. (DOCX) [file pone.0308698.s006.docx]

**S2 Table:** The first 20/88 evolutionary models for 604 Geometridae species. The best model is the one with lowest AIC score at the top as generated from IQtree model selection

| No. | Model | LnL | df |  | AIC | AICc | BIC |
| --- | --- | --- | --- | --- | --- | --- | --- |
| 1 | GTR+F+I+G4 | 72309.82 | 1221 |  | 147061.6 | 3131186 | 152566.8 |
| 2 | TIM+F+I+G4 | 72439.2 | 1219 |  | 147316.4 | 3121676 | 152812.6 |
| 3 | TIM2+F+I+G4 | 72562.05 | 1219 |  | 147562.1 | 3121922 | 153058.3 |
| 4 | TVM+F+I+G4 | 72921.42 | 1220 |  | 148282.8 | 3127523 | 153783.5 |
| 5 | SYM+I+G4 | 72954.77 | 1218 |  | 148345.5 | 3117830 | 153837.2 |
| 6 | GTR+F+G4 | 73010.2 | 1220 |  | 148460.4 | 3127700 | 153961.1 |
| 7 | TIM3+F+I+G4 | 73033.74 | 1219 |  | 148505.5 | 3122865 | 154001.7 |
| 8 | K3Pu+F+I+G4 | 73123.76 | 1218 |  | 148683.5 | 3118168 | 154175.2 |
| 9 | TIM+F+G4 | 73126.09 | 1218 |  | 148688.2 | 3118172 | 154179.9 |
| 10 | TPM2+F+I+G4 | 73171.78 | 1218 |  | 148779.6 | 3118264 | 154271.2 |
| 11 | TPM2u+F+I+G4 | 73171.84 | 1218 |  | 148779.7 | 3118264 | 154271.4 |
| 12 | TN+F+I+G4 | 73206.89 | 1218 |  | 148849.8 | 3118334 | 154341.5 |
| 13 | TIM2+F+G4 | 73248.99 | 1218 |  | 148934 | 3118418 | 154425.7 |
| 14 | TIMe+I+G4 | 73417.28 | 1216 |  | 149266.6 | 3109011 | 154749.2 |
| 15 | SYM+G4 | 73665.55 | 1217 |  | 149765.1 | 3114377 | 155252.3 |
| 16 | TVMe+I+G4 | 73682.68 | 1217 |  | 149799.4 | 3114411 | 155286.5 |
| 17 | TIM3+F+G4 | 73692.57 | 1218 |  | 149821.1 | 3119305 | 155312.8 |
| 18 | TVM+F+G4 | 73718.4 | 1219 |  | 149874.8 | 3124235 | 155371 |
| 19 | TPM3u+F+I+G4 | 73728.66 | 1218 |  | 149893.3 | 3119377 | 155385 |
| 20 | TPM3+F+I+G4 | 73728.66 | 1218 |  | 149893.3 | 3119377 | 155385 |
