# Supplementary material for: Phylogenetic structure of moth communities (Geometridae, Lepidoptera) along a complete rainforest elevational gradient in Papua New Guinea
Source: PLoS One. 2024 Aug 12;19(8):e0308698. doi: 10.1371/journal.pone.0308698 (PMC11318904; doi:10.1371/journal.pone.0308698)
Supplement: S3 Table — The moth communities with significant clustering are bolded. SES.PD, NRI and NTI are visualized as Fig 3 in the main text. (DOCX) [file pone.0308698.s007.docx]

**S3 Table:** The *picante* run summary table for the phylogenetic structure in SES.PD, the mean pairwise distance (MPD) and its derived NRI and the mean nearest taxon distance (MNTD) and its derived NTI. The moth communities with significant clustering are bolded. SES.PD, NRI and NTI are visualized as Fig 3 in the main text.

| Phylo.metric | Elevation | ntaxa | pd.obs | pd.rand.mean | pd.rand.sd | pd.obs.rank | pd.obs.z | pd.obs.p | runs |  |
| --- | --- | --- | --- | --- | --- | --- | --- | --- | --- | --- |
| SES.PD | **200** | **179** | **98.104** | **102.250** | **1.865** | **22** | **-2.224** | **0.022** | **1000** |  |
|  | 700 | 198 | 109.251 | 110.896 | 1.873 | 194 | -0.879 | 0.194 | 1000 |  |
|  | 1200 | 283 | 149.203 | 147.835 | 1.983 | 747 | 0.690 | 0.746 | 1000 |  |
|  | 1700 | 289 | 147.493 | 150.181 | 1.989 | 101 | -1.351 | 0.101 | 1000 |  |
|  | **2200** | **232** | **120.959** | **126.027** | **1.954** | **5** | **-2.594** | **0.005** | **1000** |  |
|  | **2700** | **156** | **81.648** | **91.228** | **1.764** | **1** | **-5.431** | **0.001** | **1000** |  |
|  | **3200** | **45** | **27.553** | **31.856** | **0.972** | **1** | **-4.426** | **0.001** | **1000** |  |
|  | **3700** | **8** | **5.885** | **6.858** | **0.313** | **8** | **-3.109** | **0.008** | **1000** |  |
|  | Elevation | ntaxa | mpd.obs | mpd.rand.mean | mpd.rand.sd | mpd.obs.rank | mpd.obs.z | mpd.obs.p | runs | NRI |
| MPD | 200 | 179 | 1.828 | 1.830 | 0.010 | 391 | -0.208 | 0.391 | 1000 | 0.208 |
|  | 700 | 198 | 1.831 | 1.830 | 0.009 | 512 | 0.081 | 0.511 | 1000 | -0.081 |
|  | 1200 | 283 | 1.833 | 1.831 | 0.007 | 603 | 0.308 | 0.602 | 1000 | -0.308 |
|  | **1700** | **289** | **1.812** | **1.830** | **0.006** | **2** | **-2.813** | **0.002** | **1000** | **2.813** |
|  | **2200** | **232** | **1.807** | **1.831** | **0.008** | **2** | **-3.046** | **0.002** | **1000** | **3.046** |
|  | **2700** | **156** | **1.813** | **1.830** | **0.010** | **48** | **-1.657** | **0.048** | **1000** | **1.657** |
|  | 3200 | 45 | 1.829 | 1.831 | 0.022 | 424 | -0.096 | 0.424 | 1000 | 0.096 |
|  | **3700** | **8** | **1.661** | **1.830** | **0.064** | **18** | **-2.628** | **0.018** | **1000** | **2.628** |
|  | Elevation | ntaxa | mntd.obs | mntd.rand.mean | mntd.rand.sd | mntd.obs.rank | mntd.obs.z | mntd.obs.p | runs | NTI |
| MNTD | **200** | **179** | **0.862** | **0.931** | **0.028** | **6** | **-2.510** | **0.006** | **1000** | **2.510** |
|  | 700 | 198 | 0.876 | 0.908 | 0.027 | 116 | -1.211 | 0.116 | 1000 | 1.211 |
|  | 1200 | 283 | 0.840 | 0.833 | 0.020 | 634 | 0.352 | 0.633 | 1000 | -0.352 |
|  | 1700 | 289 | 0.803 | 0.829 | 0.020 | 99 | -1.318 | 0.099 | 1000 | 1.318 |
|  | **2200** | **232** | **0.826** | **0.876** | **0.024** | **10** | **-2.110** | **0.010** | **1000** | **2.110** |
|  | **2700** | **156** | **0.823** | **0.963** | **0.031** | **1** | **-4.595** | **0.001** | **1000** | **4.595** |
|  | **3200** | **45** | **0.929** | **1.234** | **0.066** | **1** | **-4.609** | **0.001** | **1000** | **4.609** |
|  | **3700** | **8** | **1.284** | **1.560** | **0.127** | **29** | **-2.181** | **0.029** | **1000** | **2.181** |
