# Supplementary material for: Phylogenetic structure of moth communities (Geometridae, Lepidoptera) along a complete rainforest elevational gradient in Papua New Guinea
Source: PLoS One. 2024 Aug 12;19(8):e0308698. doi: 10.1371/journal.pone.0308698 (PMC11318904; doi:10.1371/journal.pone.0308698)

**S4 Table:** The table of second order polynomial regression of phylogenetic matrices to plant species richness, predator abundance and mean temperature as predictors for Geometridae moth phylogenetic structure. The null models (all indicating significance) represented by intercept are highlighted grey while other significant models are bolded.


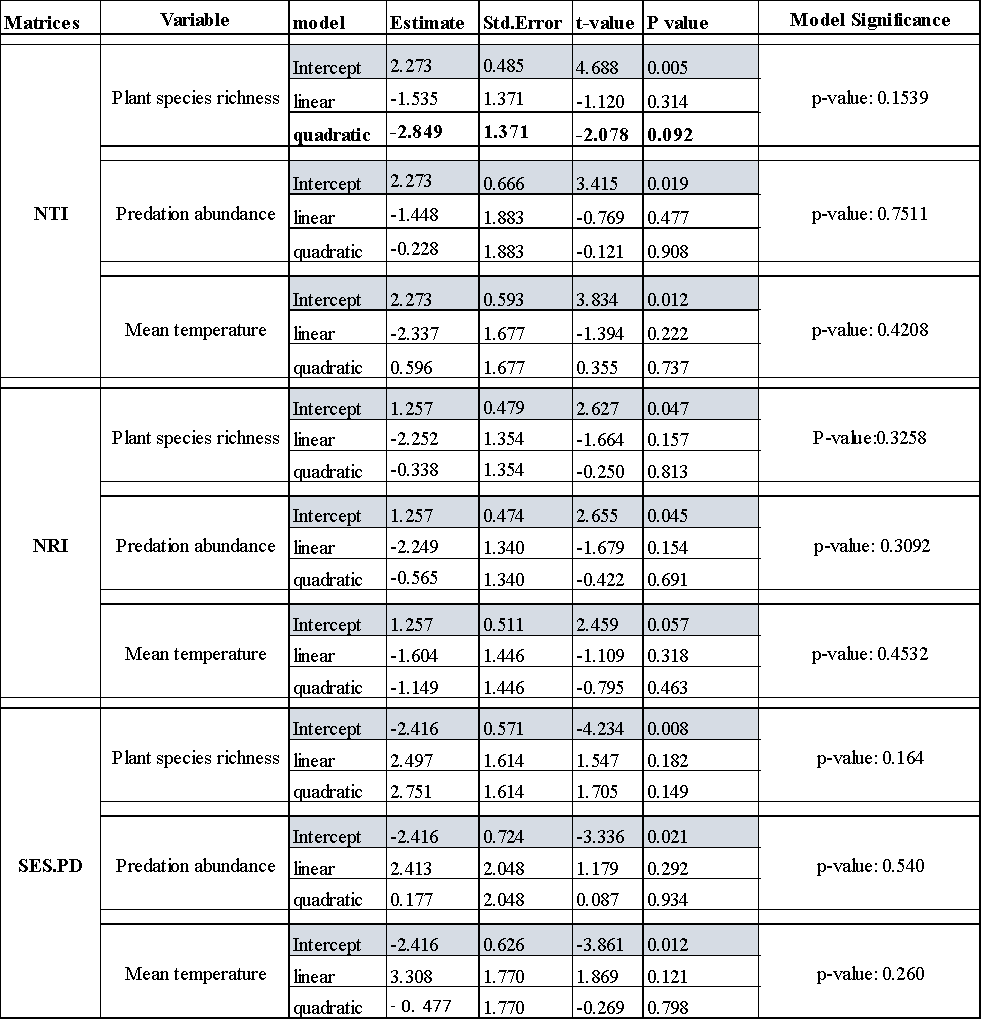

Supplement: S4 Table — The null models (all indicating significance) represented by intercept are highlighted grey while other significant models are bolded. (DOCX) [file pone.0308698.s008.docx]
