# Supplementary material for: Phylogenetic structure of moth communities (Geometridae, Lepidoptera) along a complete rainforest elevational gradient in Papua New Guinea
Source: PLoS One. 2024 Aug 12;19(8):e0308698. doi: 10.1371/journal.pone.0308698 (PMC11318904; doi:10.1371/journal.pone.0308698)
Supplement: Attachment — Submitted filename: Responses to reviewers.docx [file pone.0308698.s013.docx]

**24 July 2024**

Dear Dr. Mostafa Ghafouri Moghaddam

Academic editor

PLOSONE

**Re: Resubmission of the revised manuscript No. ‘PONE-D-24-23196’.**

Thank you for reviewing our manuscript titled "Phylogenetic structure of moth communities (Geometridae, Lepidoptera) along a complete rainforest elevational gradient in Papua New Guinea" and offering us minor revisions. We have carefully addressed the reviewers’ comments, corrections and suggestions, and below, we provide point-by-point responses to each of them. The revised manuscript is attached as a marked-up copy that highlights changes made to the original version, and an unmarked version of the manuscript without tracked changes. The lines indicated in the responses refers to those in the unmarked manuscript.

We hope that the revised version satisfies your expectations and meets the journal’s requirements.

Your sincerely,

Sentiko Ibalim (Corresponding author).

# **Responses to the reviewers.**

## **Reviewer # 1**

**Applausal 1**

The paper is well written, addresses all the relevant literature and is based on extensive samples that have been worked out through a combination of morphological and molecular taxonomy.

We thank the reviewer for the comment.

**Applausal 2**

The main weakness of the study is the low number of spatial replicates, which precludes any more sophisticated analyses. But given the many obstacles to be surmounted when assembling such a wonderful data set, I see that as a minor point – evidently these data will serve as a benchmark and baseline for decades to come.
I have only a handful of suggestions when revising the paper.

The low number of sites is indeed a weakness, but we have to weigh the number of sites against the sample size for each of them and chose large scale sampling at each site to create a solid data baseline for the future studies.

**Comment 1**

L 35: typo, should read Sorensen.

Corrected

**Comment 2**
L 228: perhaps better “quantifying potential resources”, since I suspect host plant relationships of most of these PNG moths remain undocumented thus far.

L35: We have changed the term to “potential” as proposed by the reviewer.

**Comment 3**

L 233: while I accept that focussing on woody plants was the only feasible way to assess vegetation diversity in such a diverse and largely inaccessible rainforest, it should be noted somewhere in the paper that this approach ignores potentially relevant larval food resources such as understory herbs, epiphytes, and perhaps in some geometrid species also litter, lichens, fungi and alike; see for example <https://doi.org/10.1017/S0266467415000243>.

This is indeed an important point, and the information is added in L237-238 and discussed in L485-487.

**Comment 4**

L 257: you mainly used phylogenetic dissimilarity matrices to partition them into turnover and nestedness components and to check for the strength of differences relative to elevation. This is all fine. However, I suggest you could also attempt to relate, using (Spearman?) matrix correlations, the spatial pattern of community differences (Phylo-Sorensen matrix) with matrices that describe the (Euclidean) distance between pairs of sites in terms of temperature, woody plant richness, and predator abundance. While you did not observe any strong relationships with regard to (phylogenetic) alpha diversity of geometrids and these 3 potential drivers, perhaps you see more pattern if you use beta diversity (more precisely: differentiation diversity) as a response variable here?

We have done this, using Mantel test for woody plants we have complete data set to examine the changes in Phylo-Sorensen dissimilarity relative to woody plant Sorensen dissimilarity in the revised manuscript. The test is significant and is added in the following lines:

Method: L278 – 282

Results: L362- 364

Discussion: L485-487

## **Reviewer #2**

**General comment**

The phylogenetic analysis was performed in an appropriate way and the presented conclusions sound reliable and well-substantiated. The discussion of the results is very accurate and detailed. The figures are well prepared and the supporting info well prepared with scientific names almost without misspellings.
I recommend accepting this paper in the present form, after correcting the very few details as following.

We thank the reviewer for the positive comments.

**Specific comments below:**

All second reviewer’s comments and suggestions below were changed accordingly.

**Suggestion 1**
Line 29: a.s.l -> a.s.l. (also in line 121, 123 a.s.o.) (alternatively „AMSL“?).

**Suggestion 2**

Line 46: altitudinal -> Altitudinal

**Suggestion 3**
Line 51: Does it really make sense to write „1.0“ here? I would recommend changing it to „1.“

**Suggestion 4**
Line 117: Does it really make sense to write „2.0“ here? I would recommend changing it to „2.“ (a.s.o.).

**Correction 1**

Line 293: please take away the dot at the end (you don’t have it in the other headlines)

**Correction 2**

Line 297: 3700m -> 3700 m (insert spaces also in lines 330, 333, 371, 373, 374, 375, 416 etc.)

**Correction 3**

Line 411: ( see -> (see

**Correction 4**

Line 422: 2700 -> 2700 m

**Correction 5**

Suppl. File 1, line 89: _cliptopera -> _Ecliptopera

**Correction 6**

Suppl. File 1, lines 330, 331: _comostola -> _Comostola
